# Editorial: Microorganisms for Consolidated 2nd Generation Biorefining

**DOI:** 10.3389/fmicb.2022.940610

**Published:** 2022-06-17

**Authors:** Soo Rin Kim, Carrie A. Eckert, Roberto Mazzoli

**Affiliations:** ^1^School of Food Science and Biotechnology, Research Institute of Tailored Food Technology, Kyungpook National University, Daegu, South Korea; ^2^Synthetic Biology Group, Biosciences Division, Oak Ridge National Laboratory, Oak Ridge, TN, United States; ^3^Structural and Functional Biochemistry, Laboratory of Proteomics and Metabolic Engineering of Prokaryotes, Department of Life Sciences and Systems Biology, University of Turin, Turin, Italy

**Keywords:** biomass pre-treatment, recombinant cellulolytic strategy, native cellulolytic strategy, metabolic engineering, cellulase, solvent tolerance, metabolic burden

In the last few decades, lignocellulosic biomass has attracted substantial interest as a feedstock for fermentative production of fuels and other commodity chemicals due to its wide availability and low cost (Sims et al., 2010). However, lignocellulose has innate complexity and recalcitrance to biodegradation. In natural environments, effective plant biomass decay is obtained by synergistic activity of complex microbial communities (Auer et al., 2017; Liu et al., 2021; Rajeswari et al., 2021). No natural cellulolytic microorganism isolated so far can efficiently produce high-value compounds at a scale required for commercialization. On an industrial level, this traditionally requires complex process configurations, namely the need for physical and/or chemical pre-treatment (to lower biomass recalcitrance) and multiple bioreactors dedicated to cellulase production and/or biomass saccharification and/or soluble sugar fermentation (Lynd et al., 2002). The requirement for multiple process steps seriously threatens economic viability of 2nd generation biorefining processes. The most challenging barriers to developing cost-sustainable lignocellulose biorefining process include: (1) the need for costly biomass pre-treatment which may additionally generate compounds that inhibit fermenting microorganisms; (2) dependence on high loads of expensive cellulase mixtures for biomass saccharification; and (3) issues in efficient co-fermentation of hexose and pentose sugars (e.g., because of carbon catabolite repression). Substantial research efforts have been devoted to develop consolidated bioprocessing (CBP) of lignocellulose to high-value products without the use of exogenous enzymes, namely single-pot fermentation. Motivation for this highly ambitious fermentative strategy is based on the dramatic reduction of process cost (i.e., 40–77%) with respect to traditional (less consolidated) configurations (Lynd et al., 2005, 2008). The studies included in this Research Topic touch on some key research areas for achieving CBP, as summarized below.

A variety of physical and/or chemical pre-treatment methods have been developed to decrease lignocellulose recalcitrance to biodegradation through separation of biomass components (e.g., cellulose, hemicellulose and lignin), improvement of accessibility to enzymes and microorganisms, and reduction of crystallinity (Zhou and Tian, 2022). However, these processes are typically cost challenging and, depending on the technology used, subject to the formation of compounds that can inhibit microbial growth. These issues, in part, have been tackled by the development of technologies with lower impact on costs and fermentation efficiency (Cagnin et al.). Other studies have been focused on the selection (Cagnin et al.) and/or engineering (Abdel-Rahman and Sonomoto, 2016) of microbial strains more resistant to pre-treatment inhibitors. Steam-explosion is among the most effective lignocellulose pre-treatment methodologies, yet may produce a number of inhibitors such as phenolic compounds, furans, and weak acids (García et al., 2014; Morales et al., 2017; Park et al., 2020). As included in this special Research Topic collection, the innate tolerance of seven natural *Saccharomyces cerevisiae* strains toward liquors derived from steam explosion of sugarcane bagasse, common reed, and cardoon was compared to that of the benchmark industrial *S. cerevisiae* strain, Ethanol Red (ER), currently utilized in 2nd generation ethanol plants (Cagnin et al.). Fermentative performances of the strain with the highest tolerance to steam-explosion liquors (namely Fm17) were then compared to those of *S. cerevisiae* ER. In growth media containing cardoon or common reed pre-hydrolysates, strain Fm17 showed ethanol yields 5–15% higher than those of reference strain ER. Within this specific research area, it is worth remembering that cost-competitive processes alternative to pre-treatment have recently been proposed to enhance biological solubilization of lignocellulosic feedstocks (Balch et al., 2017; Lynd et al., 2017). These include biomass milling during fermentation, which is commonly referred to as cotreatment (Balch et al., 2017; Lynd et al., 2017). This technology promoted switchgrass fermentation by *Clostridium thermocellum*, although it does not seem suitable for other microorganisms such as *S. cerevisiae* (Balch et al., 2017).

Development of lignocellulose CBP has been pursued through two main approaches: (i) engineering microbial strains that feature both (hemi)cellulolytic and high-value compound producing properties (Soucaille et al., 2010; Gandini et al., 2017; Tian et al., 2019; Wen et al., 2019; Mazzoli, 2020); (ii) assembly artificial microbial consortia consisting of (hemi)cellulolytic and high-value compound producing microorganisms (Wen et al., 2014; Shahab et al., 2018; Jiang et al., 2020; Lu et al., 2020; Schlembach et al., 2020). In regards metabolic engineering of recombinant microorganisms for CBP of lignocellulose, studies refer to two main paradigms, native and recombinant cellulolytic strategies (Lynd et al., 2002, 2005; Alper and Stephanopoulos, 2009). Native cellulolytic strategies focus on the introduction and/or improvement of high-value chemical production in native (hemi)cellulolytic microorganisms (e.g., *Clostridium cellulovorans, Clostridium thermocellum, Myceliophthora thermophila*) (Li et al., 2019; Mazzoli and Olson, 2020; Bao et al., 2021). Recombinant cellulolytic strategies intend to equip high-value product forming microorganisms with the ability to directly ferment (hemi)cellulose (e.g. *Yarrowia lipolytica, S. cerevisiae*) (Willson et al., 2016; Guo et al., 2018; Stern et al., 2018; Tang et al., 2018; Anandharaj et al., 2020). In addition, promising results have recently been reported by fusion of protoplasts of cellulolytic and compound-producing (i.e., butanol) microorganisms (Begum and Dahman, 2015; Syed and Dahman, 2015) or by biomass fermentation by using natural lignocellulose-degrading microbial communities such as the microbiota of herbivore rumen or termite gut (Auer et al., 2017; Liu et al., 2021; Rajeswari et al., 2021).

Recombinant cellulolytic strategies have been severely hampered by some major issues: (i) the extreme complexity and sophistication of native cellulase systems (Xu et al., 2015; Leis et al., 2017; Bule et al., 2018; Galera-Prat et al., 2020) (together with the high recalcitrance of lignocellulosic substrates) makes it difficult to mimic their efficiency through minimal artificial enzyme mixtures/complexes; (ii) insufficient understanding of the mechanisms promoting cellulase secretion (Yan and Wu, 2013, 2014; De Paula et al., 2019) as well as species-specific protein secretion mechanisms challenge rational engineering of recombinant cellulolytic strains. Heterologous expression of cellulases has frequently been associated with cell toxicity (Mingardon et al., 2011; Kovács et al., 2013; Tarraran et al., 2021), and/or cellulase proteolysis by the host (Mingardon et al., 2005, 2011), and/or low levels of cellulase activity (Van Rensburg et al., 2012) and/or the activation of the unfolded protein response (Ilmén et al., 2011), and/or metabolic burden (Ding et al., 2018). Metabolic burden refers to perturbation of host metabolism by heterologous protein expression and is generally attributed to energetic costs and competition for gene transcription/protein translation cell machinery associated with production of heterologous cellulases which generally cause a decrease in growth efficiency (Van Rensburg et al., 2012). The study by Wei et al. addressed the metabolic burden caused by co-expression of three fungal cellulases in the oleaginous yeast *Y. lipolytica*. *Y. lipolytica* has traditionally been used for industrial production of nutritional products, organic acid and erythritol, but recently has also emerged as potential biofuel cell factory (lipids, fatty alcohols). Coexpression of these enzymes in *Y. lipolytica* led to reduction of cell growth and lipid accumulation (Wei et al., 2019). Inactivation of Snf1, an AMP-activated serine threonine protein kinase that generally represses energy-demanding biosynthesis of lipids and proteins, and overexpression of genes involved in lipid biosynthesis promoted increased growth rate, lipid accumulation, and cellulase activity of the recombinant *Y. lipolytica* (Wei et al.). This strategy reported in this study included in the present Research Topic may therefore represent a general tool for improving the robustness of *Y. lipolytica* and other microbial platforms for CBP of lignocellulosic biomass.

Improvement of cellulase secretion in heterologous hosts has been pursued utilizing different approaches (Mazzoli et al., 2012; Tarraran and Mazzoli, 2018) such as engineering the cellulase signal peptide (Wieczorek and Martin, 2010; Stern et al., 2018) and/or inactivating housekeeping proteases of the host (Arai et al., 2007; Wieczorek and Martin, 2010). It is worth noting that, based on the limited understanding of cellulase secretion mechanisms (Yan and Wu, 2013, 2014; De Paula et al., 2019), the number of rational strategies to promote their export is reduced. Grafting a carbohydrate binding module (CBM3a) and one/two X2 domain(s) (whose precise function is not known) to the N-terminus of *C. cellulolyticum* Cel48F/Cel9G was able to promote their secretion in *C. acetobutylicum* (Chanal et al., 2011). However, the molecular mechanisms underlying this observation are not known. Owing to the complexity of protein secretion systems, differences among microbial species, and peculiarities and native cellulase-producing microorganisms (which can secrete very high amounts of cellulases) (You et al., 2012), most studies reported so far have been based on a trial-and-error approach in order to find the most compatible enzymes for a host (Ilmén et al., 2011; Mingardon et al., 2011). Alternatively, the study by Gronchi et al. included in the present Research Topic focuses on the potential to isolate new natural strains with enhanced protein secretion ability. Here, the yeast strain *S. cerevisiae* L20 showed superior ability to secrete heterologous α-amylase and glucoamylase. Genome analysis revealed that most variations in gene copy number in *S. cerevisiae* L20 with respect to the reference *S. cerevisiae* strain were related to membrane transporters and secretion pathway proteins (Gronchi et al.). This strain shows high potential also for enhanced secretion of other hydrolases, such as lignocellulose depolymerizing enzymes and encourages similar investigations on other microbial models.

For the viability of industrial fermentation processes using native cellulolytic host, product titer, yield, and productivity should be improved. For instance, for economic sustainability of fermentative production of carboxylic acids 50–100 g/L titer, 1–3 g/L/h productivity, and >0.5 g/g yield are generally required (Warnecke and Gill, 2005; Wang et al., 2016). However, product toxicity frequently threatens our ability to meet these parameters, especially in regards to titer and productivity, such as for n-butanol (Huang et al., 2010; Nicolaou et al., 2010; Mazzoli, 2021). n-butanol is among the chemicals which have been targeted as products from fermentation of lignocellulose, owing to its high potential as a drop-in fuel (Gu et al., 2011; Jiang et al., 2015). Because of its four-carbon chain, n-butanol has properties more similar to that of gasoline with respect to ethanol (only a two carbon chain) such as high combustion energy and low volatility and corrosivity (Dürre, 2007). However, butanol fermentation suffers from much higher butanol toxicity for microorganisms compared to ethanol (Heipieper et al., 2007). Currently, *Clostridium cellulovorans* is the most successful paradigm of butanol-pathway engineering in a cellulolytic microorganism (Wen et al., 2020; Bao et al., 2021). Unfortunately, *C. cellulovorans* can only tolerate very low butanol concentrations (up to 8 g L^−1^, i.e., ≈1% v/v) (Yang et al., 2015; Costa et al.). Detailed understanding of the mechanisms of butanol cell toxicity and microbial responses to butanol stress is a tool for developing targeted metabolic engineering strategies able to improve butanol tolerance in a microorganism. As for similar investigations on other microbial models, the proteomic analysis on butanol-challenged *C. cellulovorans* included in this Research Topic (Costa et al.) showed the complexity of cellular adaptive mechanisms triggered by solvent exposure. From a general standpoint, butanol elicits similar responses in different microorganisms, such as the so called homeoviscous adaptation (namely, a modification of the cell membrane composition to balance the increased fluidity caused by solvents), the overexpression of heat shock proteins, the downregulation of protein translation (to attenuate the effects of butanol on protein denaturation), and the adaptation of biochemical systems for pH and energy homeostasis (Costa et al.). However, a more detailed analysis reveals a number of gaps in understanding the mechanisms underpinning these observations or inconsistencies likely related to species-specificities. Therefore, a detailed global understanding of microbial response to butanol stress will be crucial to substantially improve butanol tolerance by targeted metabolic engineering and currently remains elusive. It is clear that adaptation to solvents involves the whole microbial cell, similar to responses to other major physical-chemical stresses (e.g., heat shock, pH) (Mazzoli, 2021). Genome wide engineering techniques (Si et al., 2017) or engineering global gene regulators involved in stress response (e.g., small non-coding RNAs, or RNA chaperones) (Venkataramanan et al., 2013; Jones et al., 2016; Sun et al., 2017; Liu et al., 2019, 2020; Liang et al., 2021) could be more suitable tools to developing butanol hypertolerant strains.

In summary, this Research Topic provides excellent examples to perform CBP of cellulosic biomass using native and heterologous cellulolytic hosts. With these research efforts, CBP can be a promising option to improve existing industrial facilities for the production of cost-competitive cellulosic fuels and chemicals.

## Author Contributions

All authors listed have made a substantial, direct, and intellectual contribution to the work and approved it for publication.

## Funding

SK carried out this work with the support of the “Cooperative Research Program for Agriculture Science and Technology Development (Project No. PJ01577003)” Rural Development Administration, Republic of Korea. CE acknowledges funding from The Center for Bioenergy Innovation, a U.S. Department of Energy Research Center supported by the Office of Biological and Environmental Research in the DOE Office of Science for the preparation of this manuscript. RM acknowledges support by Ricerca Locale (ex 60%) 2021 funding.

## Conflict of Interest

The authors declare that the research was conducted in the absence of any commercial or financial relationships that could be construed as a potential conflict of interest.

## Publisher's Note

All claims expressed in this article are solely those of the authors and do not necessarily represent those of their affiliated organizations, or those of the publisher, the editors and the reviewers. Any product that may be evaluated in this article, or claim that may be made by its manufacturer, is not guaranteed or endorsed by the publisher.
